# Construction of Women’s All-Around Speed Skating Event Performance Prediction Model and Competition Strategy Analysis Based on Machine Learning Algorithms

**DOI:** 10.3389/fpsyg.2022.915108

**Published:** 2022-07-12

**Authors:** Meng Liu, Yan Chen, Zhenxiang Guo, Kaixiang Zhou, Limingfei Zhou, Haoyang Liu, Dapeng Bao, Junhong Zhou

**Affiliations:** ^1^Sports Coaching College, Beijing Sport University, Beijing, China; ^2^Department of Physical Education, Nanjing University of Aeronautics and Astronautics, Nanjing, China; ^3^College of Sports, Chengdu University of Traditional Chinese Medicine, Chengdu, China; ^4^School of Strength and Conditioning Training, Beijing Sport University, Beijing, China; ^5^AI Sports Engineering Lab, School of Sports Engineering, Beijing Sport University, Beijing, China; ^6^China Institute of Sport and Health Science, Beijing Sport University, Beijing, China; ^7^Harvard Medical School, Hebrew SeniorLife Hinda and Arthur Marcus Institute for Aging Research, Boston, MA, United States

**Keywords:** machine learning, speed skating, performance prediction, elite athletes, model construction

## Abstract

**Introduction:**

Accurately predicting the competitive performance of elite athletes is an essential prerequisite for formulating competitive strategies. Women’s all-around speed skating event consists of four individual subevents, and the competition system is complex and challenging to make accurate predictions on their performance.

**Objective:**

The present study aims to explore the feasibility and effectiveness of machine learning algorithms for predicting the performance of women’s all-around speed skating event and provide effective training and competition strategies.

**Methods:**

The data, consisting of 16 seasons of world-class women’s all-around speed skating competition results, used in the present study came from the International Skating Union (ISU). According to the competition rules, distinct features are filtered using lasso regression, and a 5,000 m race model and a medal model are built using a fivefold cross-validation method.

**Results:**

The results showed that the support vector machine model was the most stable among the 5,000 m race and the medal models, with the highest AUC (0.86, 0.81, respectively). Furthermore, 3,000 m points are the main characteristic factors that decide whether an athlete can qualify for the final. The 11th lap of the 5,000 m, the second lap of the 500 m, and the fourth lap of the 1,500 m are the main characteristic factors that affect the athlete’s ability to win medals.

**Conclusion:**

Compared with logistic regression, random forest, K-nearest neighbor, naive Bayes, neural network, support vector machine is a more viable algorithm to establish the performance prediction model of women’s all-around speed skating event; excellent performance in the 3,000 m event can facilitate athletes to advance to the final, and athletes with outstanding performance in the 500 m event are more likely competitive for medals.

## Introduction

Accurately predicting the performance during the actual competition can help develop training plans and determine optimal strategies for athletes, which is extremely important to winning the competition (Ofoghi et al., 2016; Bunker and Susnjak, 2022). For example, Novak et al. developed a multiple linear regression model and predicted Olympic distance cross-country mountain biking field performance. Then the knowledge obtained from the prediction helped design appropriate training programs for the athletes in this field (Novak et al., 2018). However, studies have shown that the prediction of athletic performance is challenging because of the complicated scoring system and competition rules of the sport [e.g., all-around speed skating event (Ofoghi et al., 2016)], the requirement of the multi-modal coordination of the physiological systems in athletes (Maier et al., 2018) for the performance of the event.

Specifically, women’s all-around speed skating event consists of four successive individual subevents, namely the 500, 1,500, 3,000, and 5,000 m races. Only athletes who ranked top eight the scores in the first three events (i.e., 500, 1,500, and 3,000 m) can enter the final 5,000 m competition. The ranking is by calculating the average time of 500 meters for each event (i.e., the number of seconds the athlete costs is the number of points she scores), and the lower the score, the higher the ranking. This unique scoring system thus requires athletes to utilize different strategies of training and competitions for different goals of this event; that is, some may aim at entering in the last 5,000 m round, and then they aim at winning the medals. Therefore, an advanced prediction model is critical for women’s all-around speed skating athletes by providing estimated performance in the following rounds for each athlete (Noordhof et al., 2016). Smyth and Willemsen (2020) previously proposed to use a case-based reasoning technique to analyze the competition results of skaters under different external environmental conditions (e.g., altitude) to help athletes adjust the taxiing rhythm in time to achieve the best sports performance. However, this approach is not suitable for all-around speed skating event. The determinants of entering a 5,000 m race and winning a medal may differ, so the athlete cannot obtain appropriate competition and training advice from this prediction method. Therefore, it is highly demanded to develop a novel prediction model for this event, which will ultimately help improve the athletic performance.

This study proposed a novel prediction model based upon machine learning (ML) techniques. The ML is believed to help make better predictions and formulate more reasonable strategies by learning mass data through its algorithms (Maier et al., 2018). It has been widely used in sport sciences, including analyzing injury risk (Karnuta et al., 2020; Huang and Jiang, 2021) and athletic performance (Sarlis and Tjortjis, 2020; Huang and Jiang, 2021). Recently, studies emerged to implement ML to predict sports competition (Blythe and Király, 2016; Kholkine et al., 2021) and the formulation of strategies for competition (Ofoghi et al., 2013b; Tian et al., 2020). However, no studies have focused on predicting the performance use ML of athletes in all-around speed skating.

This study aims to explore the feasibility of using ML to predict the competition performance in all-around speed skating. Six different ML algorithms —support vector machine (SVM), logistic regression (LR), random forest (RF), K-Nearest Neighbor (KNN), naive Bayes (NB), neural network (NN)—was used here to construct a 5,000 m-race model (i.e., to enter the 5,000 m round) and a medal model (i.e., to win the medals). The performance and functionality of these models were then explicitly examined and compared.

## Materials and Methods

### Data Source and Feature Selection

The data for this study are acquired from the International Skating Union (ISU) official website (https://live.isuresults.eu/home), covering a total of 64 world-class women’s all-around speed skating competition results in 16 seasons (i.e., 2003/04–2019/20, except for the 2009/10 season). After being counted, the dataset contains 71 features (Supplementary Table S1).

First, the competition result data (mm:ss) are converted into data with s as the unit; then, the data are normalized to be limited within the interval [0, 1] to ensure the model converges against the effect of outliers. The data normalization procedure is formularized as:


$$x_{i}{}^{\prime }=\frac{x_{i}-x_{\min}}{x_{\max}-x_{\min}},\;\;i=1,2,\ldots ,n$$

When using ML algorithms for modeling, one needs first to filter out the optimal features to improve the performance of model prediction. If all features are included, it will increase the computational complexity and reduce the model performance. Hence, dimensionality reduction becomes the key to solving the problem. This paper uses the lasso regression method to screen the features of the 5,000 m race and medal models. Lasso regression combines the advantages of both ridge regression and subset selection process so that its computation results reflect the interpretability of subset selection and the stability of ridge regression (Tibshirani, 1996; Hashem et al., 2016; Alhamzawi and Ali, 2018). Lasso regression adds to the minimum sum of squares of errors. Considering the 1-norm constraint on the regression coefficient, the formula can be given as follows:


$$\left(\alpha ,\beta \right)=\arg\min\overset{n}{\underset{i=1}{\sum }}{\left(y_{i}-\alpha_{i}-X_{i}\beta \right)}^{2}\;\mathrm{sbuject to}{\left\|\beta \right\|}_{1}<t$$

Add the constraint in the above formula to get the following form:


$$\left(\alpha ,\beta \right)=\arg\min\overset{n}{\underset{i=1}{\sum }}{\left(y_{i}-\alpha_{i}-X_{i}\beta \right)}^{2}+\lambda {\left\|\beta \right\|}_{1}$$

where, *X*_*i*_ is the *i*th group of independent variables, which are row parameters; *α* and *β* are regression coefficients, and β is the column parameter, and $∥\beta {∥}_{1}$ represents the 1-norm, which is the sum of the absolute values of the elements in the parameters; *y_i_* is the value of the dependent variable of $X_{i}$; n is the size of the dataset used for regression modeling; λ and t are the parameters in different forms of lasso regression.

### Machine Learning Model Building and Verification

Six instances of the 5,000 m race prediction model and the medal prediction model are established through SVM, RF, LR, KNN, NB, and NN algorithms (Supplementary Figure S1); the output of the model is whether the athlete can enter the 5,000 competition or win a medal. The fivefold cross-validation method was used to verify the model’s performance. The specific process was splitting the dataset into five groups and assigning them each to an independent folder, four groups used as training data for building the model, and the remaining one used as test data to verify the model’s effectiveness. Then, this process was repeated five times, and each of the five verifications was used as the result only once. Then take the average of the five results to get an estimate.

Among the algorithms, SVM adopts the linear kernel function as the primary function (Linear Support Vector Classifier, LSVC), given a set of labels corresponding to the instance, $i=1,\ldots ,l,x_{i}\in R^{P},y_{i}\in \left\{-1,+1\right\}$, which solves an unconstrained loss function optimization problem $\xi \;\left(\mathrm{w;,}x_{i}\mathrm{;,}y_{i}\right)$:


$$\underset{w}{\min}=\frac{1}{2}w^{T}w+C\overset{l}{\underset{i=1}{\sum }}\xi \left(w\mathrm{;,}x_{i}\mathrm{;,}y_{i}\right)$$

The L2-SVM loss function is used in this study:


$$\xi \left(w\mathrm{;,}x_{i}\mathrm{;,}y_{i}\right)=\max{\left(1-y_{i}w^{T}x_{i},0\right)}^{2}$$

Naive Bayes adopts Gaussian Naive Bayes:


$$P\left(x_{i}|y\right)=\frac{1}{\sqrt{2\pi \sigma_{y}^{2}}}\exp\left(-\frac{{\left(x_{i}-\mu_{y}\right)}^{2}}{2\sigma_{y}^{2}}\right)$$

where $\sigma_{y}$ and $\propto_{y}$ are estimated using maximum likelihood estimation.

Logistic regression uses the L2 penalty logistic regression function:


$$\underset{w,c}{\min}\frac{1}{2}w^{T}w+C\overset{n}{\underset{i=1}{\sum }}\log\left(\exp\left(-y_{i}\left(X_{i}^{T}w+c\right)\right)+1\right)$$

The KNN function can be expressed as (Euclidean distance):


$$p_{ij}=\frac{\exp\left(-Lx_{i}-Lx_{j}{}^{2}\right)}{\sum_{k\neq i}\exp-\left(Lx_{i}-Lx_{k}{}^{2}\right)},\;p_{ii}=0$$

Uses the Bootstrap method to select n samples from the sample set and generates n classification trees to form a random forest (Breiman, 2001; Austin et al., 2013). The voting result of the classification tree determines the classification prediction result of the new data as expressed by the following formula:


$$f\left(x\right)=\arg\;\underset{Y}{\max}\overset{n}{\underset{i=1}{\sum }}I\left(h_{i}\left(X\right)=Y\right)$$

Where *h_i_* represents the basic model of a single classification tree, *Y* represents the output variable, and *I* mean the indicative function.

The neural network model uses Multi-layer perceptron (MLP). A set of training examples $\left(x_{1},y_{1}\right),\left(x_{2},y_{2}\right),\ldots ,\left(x_{n},y_{n}\right)$, $x_{i}\in R^{n}$ are given in the MLP, $y_{i}\in \left\{0,1\right\}$, one hidden layer and one hidden neuron MLP learning function.


$$f\left(x\right)=W_{2}g\left(W_{1}^{T}x+b_{1}\right)+b_{2}$$

with $W_{1}\in R^{m}$, $W_{2},b_{1},b_{2}\in R$ being the model parameters. $W_{1}\;and\;\;W_{2}$ represent the weights of the input layer and the hidden layer, respectively; $b_{1}\;and\;b_{2}$ represent the deviations added to the hidden layer and the output layer, respectively; g(·):R→R is the activation function, set by default as the hyperbolic tangent given by:


$$g\left(z\right)=\frac{e^{z}-e^{-z}}{e^{z}+e^{-z}}$$

For binary classification, f(x) yields an output value between 0 and 1 through the logic function $g\left(z\right)=1/\left(1+e^{-z}\right)$. Samples are assigned to the positive class if having an output value greater than or equal to the threshold 0.5, else to the negative class.

The algorithm and evaluation are implemented using Scikit-learn based on Python 3 (Pedregosa et al., 2011). In the training process, the main parameters of different instances of the models are adjusted. The grid search method is used to adjust the hyperparameters to find the parameter value corresponding to the highest accuracy provided that the training data exist.

### Model Evaluation

Evaluation indicators include the area under the receiver operating characteristic curve (ROC) AUC, accuracy, sensitivity, precision, and balanced F1 score. AUC is used to evaluate the discriminative ability and performance of the model. When the value of AUC is 1, it means that the model is perfect; a value of 0.5 means the deficient performance of a random classifier, i.e., the random classifier does not have any discriminative ability; a value of 0.90–1 means excellent, 0.80–0.90 good, 0.70–0.80 fair, 0.60–0.70 poor, and 0.50–0.60 failure (Bruce et al., 2020). The correct rate is the proportion of the samples judged correctly by the classifier among all samples. The higher the correct rate, the better the classifier; sensitivity is the proportion of all positive examples judged correctly by the classifier, which measures the classifier’s ability to recognize positive examples; accuracy represents the proportion of positive examples judged to be positive by the classifier; the F1 score is the weighted average of model accuracy and recall; the maximum of the four indicators is 1, the minimum is 0, and the higher the value, the better the model (Stehman, 1997). Among the results of judgment, TP = true positive, TN = true negative, FP = false positive, FN = false negatives.


$$\mathrm{Accuracy}=\frac{\mathrm{TP}+\mathrm{TN}}{\mathrm{TP}+\mathrm{TN}+\mathrm{FP}+\mathrm{FN}}$$

$$\mathrm{Precision}=\frac{\mathrm{TP}}{\mathrm{TP}+\mathrm{FP}}$$

$$\mathrm{Sensitivity}=\frac{\mathrm{TP}}{\mathrm{TP}+\mathrm{FN}}$$

$$F1=\frac{2\mathrm{TP}}{2\mathrm{TP}+\mathrm{FP}+\mathrm{FN}}$$

### Feature Weight Calculation

The present study quantifies the impact of the included features on model performance by computing weights (Li et al., 2020). To this end, the LSVC model is used in Python 3 Scikit-learn.

## Results

### Feature Selection Results of Lasso Regression

#### Feature Inclusion in the 5,000 m Competition Model

**Figure 1:**
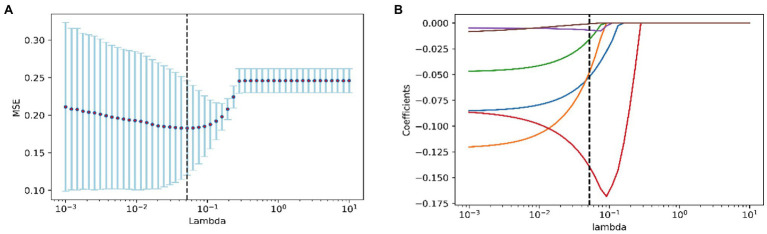
Feature screening of the 5,000 m Race Model. **(A)** The figure of test MSE by lambda value; **(B)** the path diagram of lasso regression coefficients.

Features other than those associated with the 5,000 m race were filtered using Lasso regression analysis to determine the best features to build the model. When *λ* was equal to 0.051, the model based on the following six features performed best: 3,000 m 1st lap score (3,000 m1), 3,000 m 7th lap score (3,000 m7), 1,500 m 1st lap score (1,500 m1), 3,000 m 8th split timer (3,000 ms8), 1,500 m 2nd split timer (1,500 ms^2^), and 3,000 m points (3,000 m Points; Figure 1).

#### Feature Inclusion in the Medal Model

**Figure 2:**
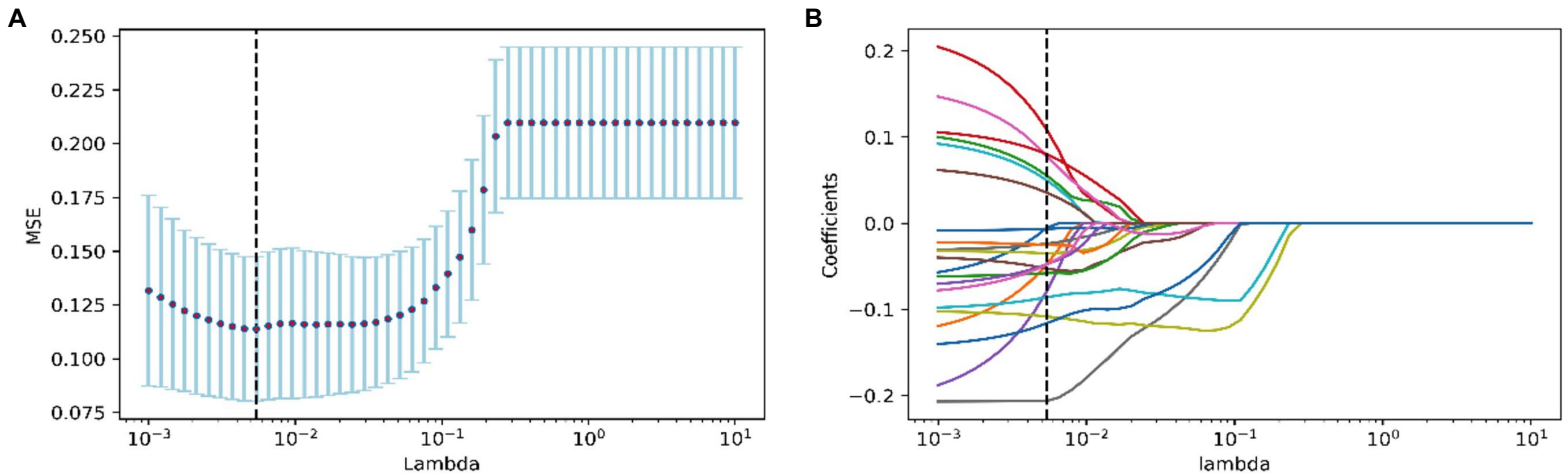
Feature screening of the Medal Model. **(A)** The figure of test MSE by lambda value; **(B)** the path diagram of lasso regression coefficients.

Lasso regression analysis was used to screen all features. When *λ* was equal to 0.0054, the model based on the following 21 features performed best: 500 m 2nd lap score (500 m2), 3,000 m 2nd lap score (3,000 m2), 3,000 m 3rd lap score (3,000 m3), 3,000 m 4th lap score (3,000 m4), 3,000 m 5th lap score (3,000 m5), 3,000 m 7th lap score (3,000 m7), 1,500 m 2nd lap score (1,500 m2), 1,500 m 3rd lap score (1,500 m3), 1,500 m 4th lap score (1,500 m4), 5,000 m 1st lap score (5,000 m1), 5,000 m 2nd lap score (5,000 m2), 5,000 m 4th lap score (5,000 m4), 5,000 m 5th lap score (5,000 m5), 5,000 m 9th lap score (5,000 m9), 5,000 m 11th lap score (5,000 m11), 5,000 m 13th lap score (5,000 m13), 5,000 m 3rd split timer (5,000 ms3), 500 m ranking, 3,000 m ranking, 1,500 m ranking, and 5,000 m ranking (Figure 2). In order to facilitate the actual operation, the 500 m ranking, 3,000 m ranking, 1,500 m ranking, and 5,000 m ranking from which features cannot be directly extracted in the test process are excluded, and the remaining 17 features were retained.

### Performance Prediction Model Results

#### Evaluation and Comparison of the 5,000 m Race Model for Women’s All-Around Speed Skating Event

**Figure 3:**
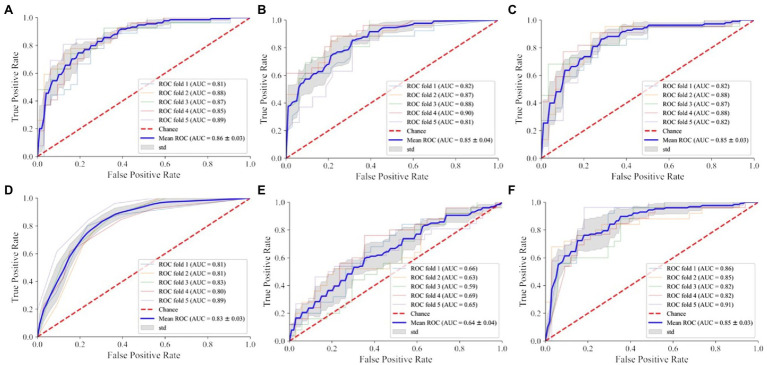
Receiver operating characteristic (ROC) curves of different models in the 5,000 m final of women’s all-around speed skating event. **(A)** SVM: support vector machine; **(B)** RF: random forest; **(C)** LR: logistic regression; **(D)** KNN: K-nearest neighbor; **(E)** NB: naive Bayes; **(F)** NN: neural network.

According to the plotted ROC curve (Figure 3), the AUC values of the six instances of the 5,000 m race model for women’s all-around speed skating event established by SVM, RF, LR, KNN, NB, and NN are 0.86, 0.85, 0.85, 0.83, 0.64, and 0.85, respectively. It can be observed that the overall better-performing algorithms are SVM, RF, LR, and NN. SVM had the most balanced classification through a comprehensive comparison of accuracy, sensitivity, and F1 score (Table 1).

**Table 1:** Validity evaluation of different prediction models for the 5,000 m final of women’s all-around speed skating event.

ML	Accuracy	Sensitivity	Precision	F1 Score
SVM	0.78 ± 0.03	0.77 ± 0.05	0.73 ± 0.04	0.75 ± 0.03
RF	0.76 ± 0.04	0.81 ± 0.06	0.67 ± 0.03	0.73 ± 0.03
LR	0.77 ± 0.04	0.76 ± 0.05	0.66 ± 0.08	0.70 ± 0.05
KNN	0.72 ± 0.01	0.71 ± 0.11	0.68 ± 0.04	0.69 ± 0.06
NB	0.62 ± 0.01	0.57 ± 0.04	0.66 ± 0.05	0.63 ± 0.04
NN	0.72 ± 0.03	0.65 ± 0.04	0.71 ± 0.04	0.68 ± 0.05

SVM, support vector machine; RF, random forest; LR, logistic regression; KNN, K-nearest neighbor; NB, naive Bayes; NN, neural network.

#### Evaluation and Comparison of the Medal Model

**Figure 4:**
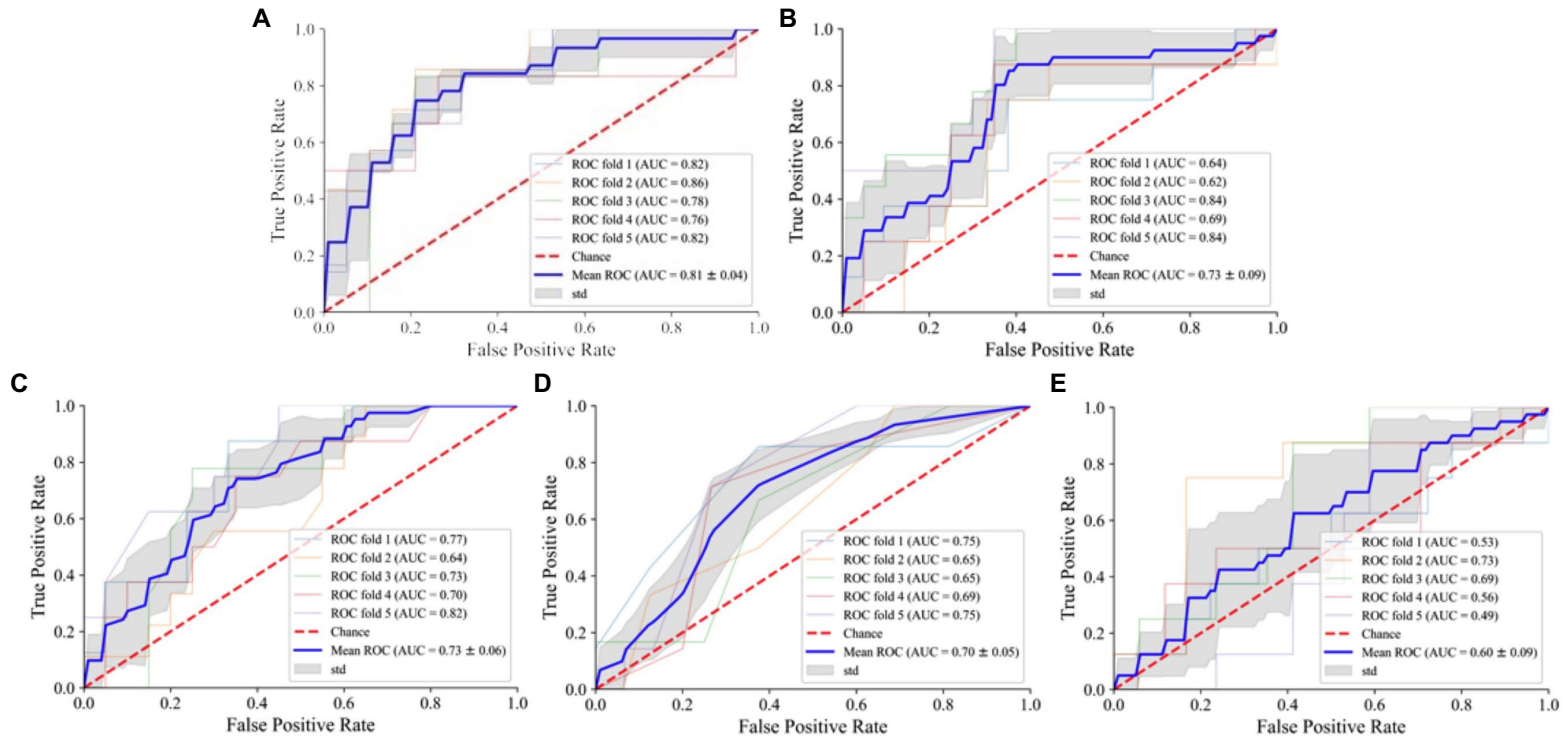
ROC curve of different models of women’s speed skating medals. **(A)** SVM: support vector machine; **(B)** RF: random forest; **(C)** LR: logistic regression; **(D)** KNN: K-nearest neighbor; **(E)** NB: naive Bayes.

In training the instances of the medal model, the NN-based instance fails due to the excess data size. According to the plotted ROC curve (Figure 4), the AUC values of the five medal events for women’s all-around speed skating event established by SVM, RF, LR, KNN, and NB are 0.81, 0.73, 0.73, 0.70, and 0.60, respectively. Among these model instances, the SVM instance proves high-performing and is the only instance that demonstrates good stability through a comprehensive comparison of accuracy, sensitivity, and F1 score of the five models (Table 2).

**Table 2:** Effectiveness of the prediction models for women’s all-around speed skating medal.

ML	Accuracy	Sensitivity	Precision	F1 score
SVM	0.80 ± 0.07	0.71 ± 0.06	0.63 ± 0.04	0.67 ± 0.08
RF	0.73 ± 0.02	0.43 ± 0.08	0.58 ± 0.02	0.49 ± 0.05
LR	0.78 ± 0.06	0.42 ± 0.08	0.8 ± 0.2	0.55 ± 0.08
KNN	0.75 ± 0.07	0.51 ± 0.06	0.63 ± 0.8	0.55 ± 0.8
NB	0.60 ± 0.07	0.59 ± 0.06	0.42 ± 0.08	0.49 ± 0.06

### Feature Weight Analysis

**Figure 5:**
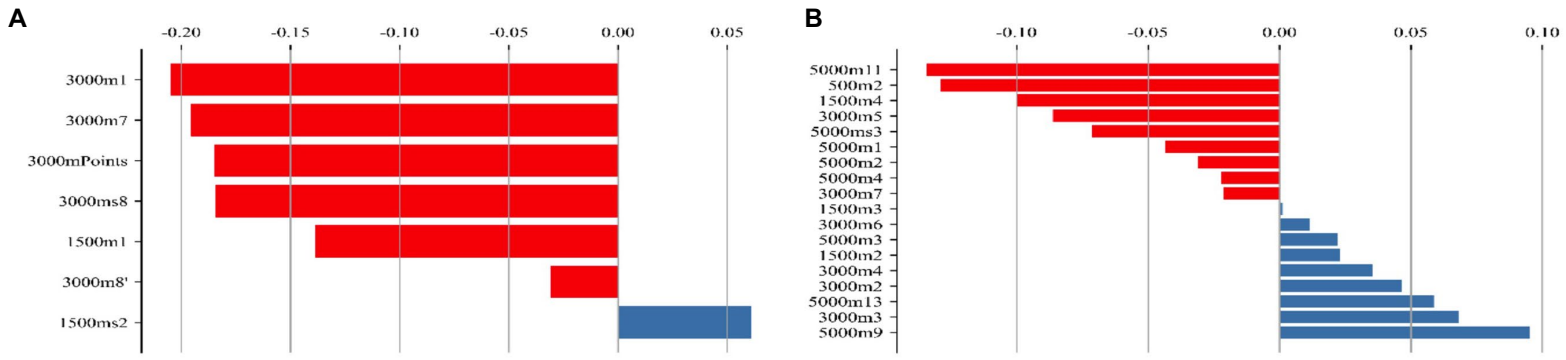
Weight of feature. **(A)** The feature weights of the 5,000 m race model; **(B)** the feature weights of the medal model.

According to the feature weights calculated by LSVC, the scores of the 3,000 m laps 1st and 7th and the individual points of the 3,000 m are the most critical features that affect whether an athlete can enter the 5,000 m competition (Figure 5A). The results of 5,000 m lap 11th, 500 m lap 2nd, and 1,500 m lap 4th are positive characteristics that affect whether athletes can win medals, while the results of 5,000 m laps 9th, 13th and 3,000 m lap 3rd are negative characteristics that affect whether athletes can win medals (Figure 5B).

## Discussion

This study examined six ML algorithms approaches based upon a real competition database of split times and ranking in women’s all-around speed skating athletes. The results have shown that it is feasible to predict the performance ranking by ML algorithms. Through comparison in the performance of the instances of the models built by different algorithms, it has been observed that the SVM-based instance can effectively predict the performance of the women’s all-around speed skating event, suggesting that this model would help athletes to set appropriate training programs, improving the quality of their strategic decision-making and competitive performance.

The model for performance prediction in women’s all-around speed skating event established through ML can provide more direct suggestions for the training and competition of this event. For instance, coaches and athletes can input the daily test results into the model to obtain the probability of athletes entering the 5,000 m competition or winning medals to help athletes and coaches in training better. This is more generic than Smyth’s (Smyth and Willemsen, 2020) use of specific case-based reasoning. The selection of features is the key to building this more general model (Horvat et al., 2020). Research has shown that lasso regression has advantages over traditional Stepwise Regression methods in feature selection (Yarkoni and Westfall, 2017). Applied for feature screening in this study, the lasso regression method is more conducive to eliminating unimportant related features and accurately screening out relatively important ones. Combined with weight calculation, the model can be more accessible and interpretable. In this study, the features of the 5,000 m race model and the medal model are distinct. In deciding whether athletes are eligible for entering the 5,000 m competition, the 3,000 m score has the most relevant features, while in determining whether the final result suffices to win a medal, things are different. Laps 1, 2, 4, 7, 11 at 5,000 m, 5, 7 laps at 3,000 m, lap 2 at 500 m, and before 3,000 m, the speed of the three laps has an important influence on whether the athlete can win a medal. This reminds coaches that the training emphasis of athletes should be highlighted for different competition purposes. If the athlete’s goal is to enter the 5,000 m race, she should first develop the long-distance racing ability until scoring high enough in the 3,000 m race for entering the 5,000 m race. However, if the athlete’s goal is to win a medal, she should also pay attention to the development of speeding ability. Athletes must not have apparent shortcomings; otherwise, the final ranking will probably be affected by the 500 m score. Athletes with outstanding 500 m scores are easier to win a medal. Moreover, one can notice that the medal winners of laps 9 and 13 of the 5,000 m race do not outspeed the non-winners. This seems to reveal that having faster speed in the first half of the 5,000 m race can be more conducive to good results. Previous studies have reported that active start-up acceleration and forward speed are conducive to achieving better athletic performance (Muehlbauer et al., 2010). This revelation also provides a reference for athletes to formulate competitive strategies. Previous studies have also shown that the decrease in the second half of the competition speed may increase the push-off angle associated with fatigue (Noordhof et al., 2013). Therefore, improving the technical stability of athletes in a fatigued state is crucial to improving sports performance. This also provides a particular idea for the election of athletes. When all-around speed skaters are elected among women athletes, sufficient attention should be paid to those with excellent aerobic capacity and explosive power.

Since modeling in this study aims to determine the probability of athletes entering the finals and winning medals, six classification algorithms were selected when each model was established. The most commonly used ML algorithms in sports include SVM, RF, LR, KNN, NB, and NN (Horvat et al., 2020). Some of these algorithms have been applied to predict the performance of some events. For example, Ofoghi et al., (2013a) used *K*-means combined with traditional statistical methods to model the performance prediction of athlete election after the all-around track cycling competition system was changed, which effectively helped coaches elect athletes and develop the appropriate training plan. The research by Dwyer et al. found that the triathlon performance prediction model based on the NB algorithm is also effective. This model helps coaches and athletes formulate reasonable competitive strategies to optimize athletes’ sports performance (Ofoghi et al., 2016). In addition, other researchers have also used different ML algorithms for performance prediction in different events (Richter et al., 2021). In this study, ML proves effective and feasible in predicting the performance ranking in women’s all-around speed skating event by learning from past competition data and establishing a viable model. Theoretically, these six models can predict performance, but the comparison has revealed differences in the predicted performance between the prediction models built on different ML algorithms. For the 5,000 m final prediction model, the AUC values of SVM, RF, LR, KNN, and NN are similar. The SVM-based instance model has achieved the best overall performance, while the AUC value of the NB-based instance is only 0.64, and its accuracy, sensitivity, and F1 score are also low (Table 1). For the medal model, the SVM-based instance of the medal model has also shown a good performance (Table 2), while the NB-based instance has performed relatively poorly, and the NN-based instance has failed.

The above differences may be ascribed to the characteristics of different algorithms. Based on conditional probability, the NB algorithm uses Bayes’ theorem to calculate the probability by determining the combination of the frequency and the historical data values. It also rests on the assumption of a given output and that the interclass attributes are independent, but this assumption is difficult to hold in practice (Rish, 2001). The same is true in this study. The NN algorithm has very high requirements on data size, which may be the reason for not being able to establish the medal model. The SVM is highly applied in solving relatively small sample predictions and is more sensitive to data. Given the relatively small dataset in this study, the final decision function of SVM has been determined by only a few support vectors. The computational complexity depends on the number of support vectors rather than on the dimensionality of the sample space, and the direct association between the input variables in this study avoids the “curse of dimensionality” in some sense (Shalev-Shwartz et al., 2011). The SVM algorithm is widely used in the domain of sports. For example, the maximum oxygen uptake prediction model established by the SVM algorithm has good prediction accuracy (Abut and Akay, 2015), the gait diagnosis model established by Begg et al. through SVM is also of high applied value (Begg et al., 2005), and the Chinese Super League ranking model built on the SVM algorithm also has high accuracy (Li et al., 2020). From the results of this research, the SVM algorithm is also feasible for performance prediction. The NB algorithm has shown application prospects for predicting the performance of complex events in previous studies, such as all-around track cycling (Ofoghi et al., 2013a), triathlon (Ofoghi et al., 2016), decathlon (Trevor et al., 2002). However, because the NB algorithm assumes that the sample attributes are independent, its effect is not satisfactory when the sample attributes are correlated. In this study, the included features may have a strong correlation, such that the NB algorithm becomes less suitable for the prediction model. NN is considered an excellent ML algorithm, but the model has poor interpretability due to the extremely high data requirements and the “black box” problem. Still, the research results show that NN does not necessarily outperform other ML algorithms in performance prediction (Bunker and Susnjak, 2022).

To sum up, the present work is that it provides information that can be used to predict future performances in women’s all-around speed skating with a certain level of accuracy. The mathematical models that form the basis for these predictions were developed from an analysis of historical race data. We believe that our analytical approach is reasonable to be confident about the accuracy of our results. Although we have performed a great deal of work, this study still had some limitations. First, this research has overfitted the available data when using NN to build the medal prediction model due to the relative lack of data. In the future, with the increase in data size, neural networks will be helpful in prediction. Secondly, ignoring the different event settings, this study failed to explore men’s all-around speed skating event. Future research can conduct a comparative study between men’s and women’s events.

## Conclusion

The ML algorithm has proven feasible in predicting women’s all-around speed skating competition performance. The prediction model built on SVM has proven more suitable for predicting women’s all-around speed skating competition performance compare with LR, RF, KNN, NB, and NN. Female speed skaters with excellent results in the 3,000 m race are entitled to enter the all-around final, while athletes with outstanding results in the 500 m race are strong competitors for a medal.

## Data Availability Statement

The original contributions presented in the study are included in the article/supplementary material, further inquiries can be directed to the corresponding authors.

## Author Contributions

KZ, DB, ML, and JZ: design and/or conceptualization of the study. KZ, ML, YC, LZ, JZ, and DB: analysis and/or interpretation of the data. KZ, JZ, and DB: drafting and/or revising the manuscript. All authors contributed to the article and approved the submitted version.

## Funding

This study was supported by the National Key Research and Development Program of China (Grant Numbers 2018YFC2000602 and 2019YFF0301803).

## Conflict of Interest

The authors declare that the research was conducted in the absence of any commercial or financial relationships that could be construed as a potential conflict of interest.

## Publisher’s Note

All claims expressed in this article are solely those of the authors and do not necessarily represent those of their affiliated organizations, or those of the publisher, the editors and the reviewers. Any product that may be evaluated in this article, or claim that may be made by its manufacturer, is not guaranteed or endorsed by the publisher.
