# Supplementary material for: Construction of Women’s All-Around Speed Skating Event Performance Prediction Model and Competition Strategy Analysis Based on Machine Learning Algorithms
Source: Front Psychol. 2022 Jul 12;13:915108. doi: 10.3389/fpsyg.2022.915108 (PMC9326501; doi:10.3389/fpsyg.2022.915108)
Supplement: Supplementary file 1 [file Table_1.DOCX]

Supplementary Material

# Supplementary Table

**Supplementary Table S1. Characteristics of the Raw Data.**

| Category | Features | Feature type |
| --- | --- | --- |
| Players | Nationality, name | classification |
| Grades, ranking | 500m score, 500m ranking, 1500m score, 1500m ranking, 3000m score, 3000m ranking, 5000m score, 5000m ranking, total ranking | continuous |
| Single lap score, split score | 500m × 4，1500m × 8，3000m × 16，5000m × 26 | continuous |
| Integral | Single points × 4, total points, points difference | continuous |
| Category | Features | Feature type |
| Players | Nationality, name | classification |
| Grades, ranking | 500m score, 500m ranking, 1500m score, 1500m ranking, 3000m score, 3000m ranking, 5000m score, 5000m ranking, total ranking | continuous |

## Supplementary Figures


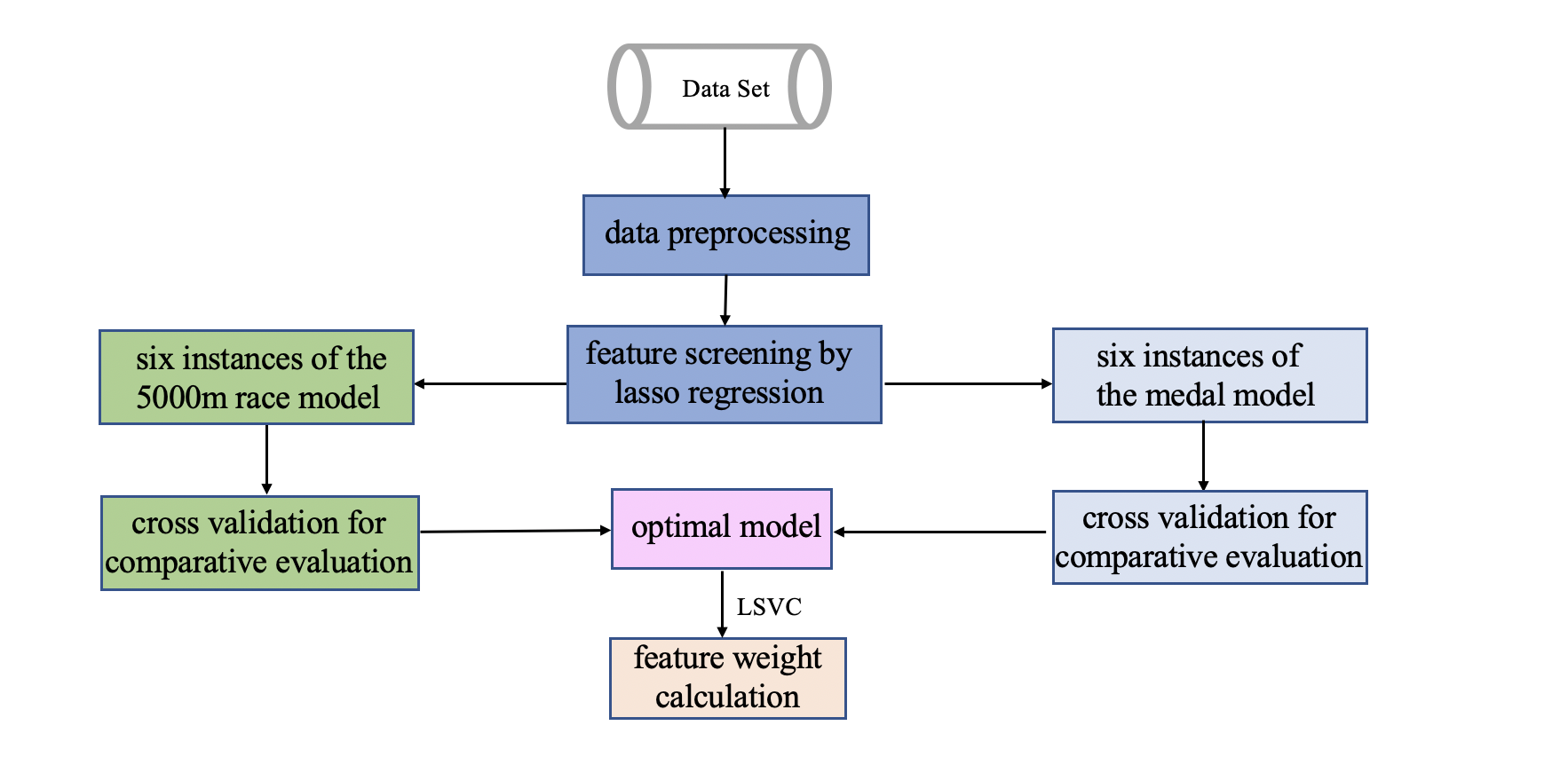


**Figure S1. The Protocol of Experiments.**
